# Facile Preparation of High-Performance Polythiophene Derivative and Effect of Torsion Angle Between Thiophene Rings on Electrochromic Color Change

**DOI:** 10.3390/molecules29225477

**Published:** 2024-11-20

**Authors:** Qingfu Guo, Chao Sun, Yiran Li, Kaoxue Li, Xishi Tai

**Affiliations:** 1 College of Chemistry and Chemical Engineering, Weifang University, Weifang 261061, China; liyr1987@foxmail.com (Y.L.); likaoxue@163.com (K.L.); 2 Shandong Peninsula Engineering Research Center of Comprehensive Brine Utilization, Weifang University of Science and Technology, Weifang 262700, China; kdsunchao@163.com

**Keywords:** poly(3,3′-dimethyl-2,2′-bithiophene), electrochromism, spatial plane distortion, electrochromic device

## Abstract

The electrochromic phenomenon of conducting polymer is mainly dominated by the π-π* band transition. The π conjugation is influenced by the coplanarity between polymer units, deviations from which can lead to an increased ionization potential and band gap values. In order to investigate the effect of plane distortion angle on electrochromic color in the main chain structure of polymerization, high-performance poly(3,3′-dimethyl-2,2′-bithiophene) (PDMeBTh) with a large plane distortion angle is successfully synthesized in boron trifluoride diethyl etherate (BFEE) by the electrochemical anodic oxidation method. The electrochemical and thermal properties of PDMeBTh prepared from BFEE and ACN/TBATFB are compared. The electrochromic properties of PDMeBTh are systematically investigated. The PDMeBTh shows a different color change (orange-yellow in the neutral state) compared to poly (3-methylthiophene) (light-red in the neutral state) due to the large torsion angle between thiophene rings of the main polymer chain. The optical contrast, response time, and coloring efficiency (CE) of the prepared PDMeBTh are also studied, which shows good electrochromic properties. For practical applications, an electrochromic device is fabricated by the PDMeBTh and PEDOT. The color of the device can be reversibly changed between orange-yellow and dark blue. The light contrast of the device is 27% at 433 nm and 61% at 634 nm. The CE value of the device is 403 cm^2^ C^−1^ at 433 nm and 577 cm^2^ C^−1^ at 634 nm. The constructed device also has good open circuit memory and electrochromic stability, showing good potential for practical applications.

## 1. Introduction

An electrochromic device (ECD) refers to an electrochemical device that has a variety of color changes under different potentials. Recently, ECDs have been widely used in smart windows, electromagnetic switches, and displays [1,2,3]. In order to improve the application space of ECDs, it is required that the device must have a fast conversion time, multiple color changes, high contrast, and good stability [4,5]. Organic conducting polymers are the preferred electrochromic materials due to their fast conversion time, high optical contrast, and good stability [6,7]. Polythiophene and its derivatives have become very valuable conducting polymer electrochromic materials, which have been applied in microelectronics and optoelectronic devices [8], electrochromic devices [9], and energy storage devices [10].

In recent studies, the electrochemical polymerization of thiophene derivatives with different substituents and their electrochromic applications have been reported [11,12]. For example, the polymerization and application of 3-substituted thiophenes, such as 3-alkylthiophene, 3-bromothiophene, 3-alkoxythiophene, etc., have been studied by researchers [13]. Among them, poly(3-methylthiophene) with promising electrochromic characteristics is an outstanding representative. Previous studies have shown that poly(3-methylthiophene) can change reversibly between blue and red under different potentials, showing good electrochromic properties [14].

**Scheme 1:**
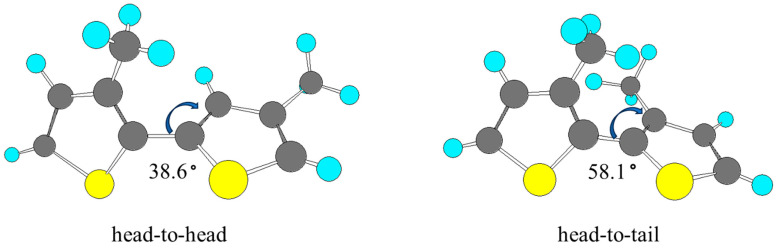
The structure of 3,4′-dimethyl-2,2′-bithiophene (head-to-head) and 3,3′-dimethyl-2,2′-bithiophene (head-to-tail).

In the process of electrochemical polymerization, the use of bithiophene instead of thiophene derivatives as the starting monomer can effectively reduce the oxidation potential, avoiding possible overoxidation and enhancing the quality of prepared polymer [15]. For the preparation of poly(3-methylthiophenes) using methyl-substituted 2,2′-bithiophenes, the monomer structure exhibits a different substituent pattern along the bithiophene backbone, and therefore, its analogues can be considered formally as “head-to-head” and “head-to-tail” coupled analogues as depicted schematically in Scheme 1. For a “head-to-head” structure (3,4′-dimethyl-2,2′-bithiophene), the prepared polymer is identical to the polymer prepared by the monomer of 3-methylthiophene. Bottoni’s group have calculated that the inter-ring dihedral angle of 3,4′-dimethyl-2,2′-bithiophene is 38.6° by the density functional theory (B3LYP) [16]. However, for a “head-to-tail” structure (3,3′-dimethyl-2,2′-bithiophene (DMeBTh)), in fact, the methyl chain can cause the main chain to twist out of the plane at a larger angle due to steric hindrance. The inter-ring dihedral angle of DMeBTh is calculated to be 58.1° by the density functional theory [16]. The electrochromic phenomenon of a conducting polymer is mainly dominated by the π-π* band transition, which depends on the overlap between the π orbitals, and thus on the degree of π conjugation along the polymer chain. Hence, the π-π conjugation is strongly influenced by the coplanarity between polymer units, deviations from which can lead to an increase in ionization potential and band gap values. Moreover, defects in a semicrystalline crystal lattice can be caused by the distorted biaryl π-system, which can affect the charge carrier mobility of the polymer. To solve this problem, Zade’s group synthesized a new polymer comprising alternate thiophene and didodecyloxymethyl-substituted cyclopenta[c]thiophene units. The designed polymer with the cyclopentane substitution maintained its planarity, thus exhibiting a good field effect mobility [17]. Therefore, for poly(3,3′-dimethyl-2,2′-bithiophene) (PDMeBTh), the large torsion angle present along the main chain structure can result in a rotational defect, which interrupts and weakens the degree of π conjugation. This reduced π conjugation may have an effect on the range of light absorption, which in turn causes it to exhibit a different color change compared with poly(3-methylthiophene). So far, no studies have been reported on the electrochromic color change in a conducting polymer due to the polymer backbone being twisted out of plane.

Solvents are very important in electrochemical polymerization reactions to prepare high-quality polymer materials [18,19,20]. Previous studies have proven that boron trifluoride diethyl etherate (BFEE) can facilitate the formation of high-quality conducting polymer films due to the interactions between BFEE and the aromatic monomers, in which the oxidation potential of the monomer will be significantly reduced [21]. In the electrochemical polymerization process, BFEE can be used as both a solvent and an electrolyte, and no other supporting electrolyte is needed [22]. Therefore, compared with traditional acetonitrile/supporting electrolyte systems, BFEE has advantages in the preparation of high-performance conducting polymers. In this study, PDMeBTh was prepared in a facile manner in BFEE by the direct anodic oxidation method. The electrochemical property and thermal stability of PDMeBTh prepared from BFEE and ACN/TBATFB were compared. The electrochromic properties of PDMeBTh prepared from BFEE were studied systematically, which shows a different color change compared with poly(3-methylthiophenes) due to the effect of torsion angle between thiophene rings.

## 2. Results and Discussion

### 2.1. Electrochemical Property of PDMeBTh

**Figure 1:**
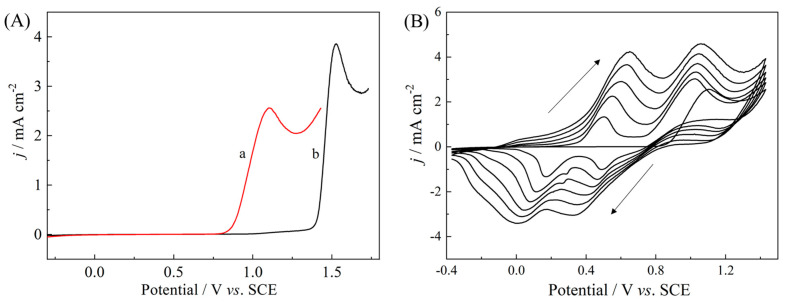
(**A**) LSV curves of DMeBTh in (a) BFEE and (b) ACN/TBATFB; (**B**) CVs of DMeBTh in BFEE.

Different polymerization systems can lead to different oxidation potentials for monomers, which will affect the quality of polymers. Figure 1A shows the linear scanning voltammetry (LSV) curves of 0.05 mol L^−1^ DMeBTh in BFEE (A) and ACN/TBATFB (B). The oxidation potential of DMeBTh in BFEE is 0.86 V vs. SCE, which is lower than the 1.39 V vs. SCE measured by ACN/TBATFB. It is also lower than the initial oxidation potential of 3-methylthiophene in BFEE (1.20 V vs. SCE) [23]. Figure S1 shows the photographs of PDMeBTh obtained in BFEE (A) and ACN/TBATFB (B) under the 1.0 V voltage, which are polymerized on the ITO electrode. It can be seen that the amount of polymer prepared in BFEE is more under the same polymerization conditions, which may be attributed to the fast reaction rate provided by BFEE over the oxidation voltage. More importantly, the polymer film prepared in BFEE is more uniform, which is very conducive to the construction of electrochromic devices. This result suggests that the oxidation of DMeBTh in BFEE is easier than in ACN/TBATFB. At the same time, DMeBTh is more easily polymerized in BFEE than 3-methylthiophene, which is more conducive to the preparation of high-performance polymers.

Figure 1B shows the CV scanning curve of 0.05 mol L^−1^ DMeBTh in BFEE. It can be seen that the CV curve in BFEE exhibits similar characteristics to other conducting polymers with wide redox peaks. This indicates that the PDMeBTh has a reversible redox process, and there are two pairs of redox peaks in the −0.37 V vs. SCE to 0.76 V vs. SCE and −0.06 V vs. SCE to 1.23 V vs. SCE regions. It is noteworthy that the redox peak current density gradually increases during the scanning process, which indicates the increase in the amount of polymer generated on the electrode [23]. This result indicates that the PDMeBTh can be successfully prepared in BFEE by the electrochemical polymerization method.

**Figure 2:**
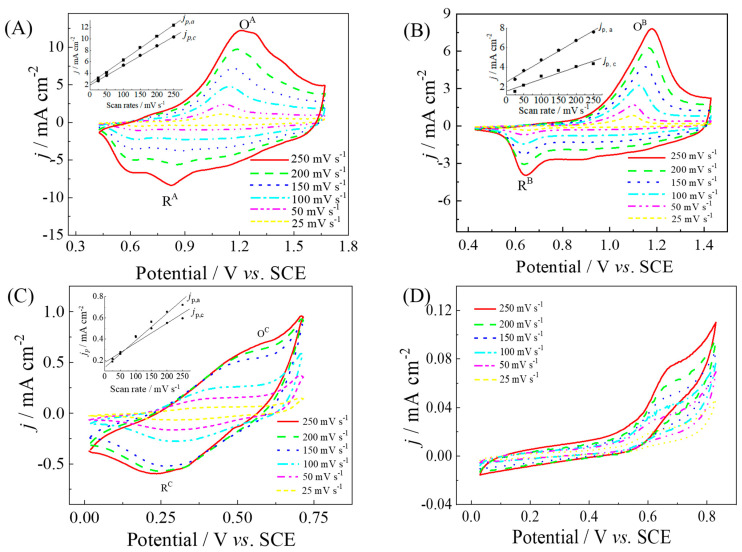
CV curves of PDMeBTh in (**A**,**B**) ACN/TBATFB and (**C**,**D**) concentrated sulfuric acid under scan rates of 25, 50, 100, 150, 200, and 250 mV s^−1^; the PDMeBTh was prepared in (**A**,**C**) BFEE and (**B**,**D**) ACN/TBATFB.

The electrochemical properties of PDMeBTh film prepared in BFEE and ACN/TBATFB systems were studied in ACN/TBATFB and concentrated sulfuric, respectively. The PDMeBTh prepared in BFEE shows steady-state CV characteristics and wide redox peaks in monomer-free ACN/TBATFB (Figure 2A). The peak current density is proportional to the scanning rate (Figure 2A, inset), which indicates that the polymer has reversible redox properties. And the prepared PDMeBTh can be reversibly redox between 0.60 vs. SCE (Ea) and 0.25 V vs. SCE (Ec) in concentrated sulfuric acid (Figure 2C). However, in ACN/TBATFB (Figure 2A), a higher potential and a wider potential range (from 0.83 vs. SCE (Ea) to 1.21 V vs. SCE (Ec)) are required to oxidize and reduce the PDMeBTh. In concentrated sulfuric acid, the peak potential difference (Ea-Ec) of PDMeBTh associated with doping–dedoping reaction kinetics is 0.35 V vs. SCE, which is 0.70 V vs. SCE in ACN/TBATFB. Hence, the redox reaction of PDMeBTh is faster in concentrated sulfuric acid than in ACN/TBATFB without monomers.

The electrochemical properties of PDMeBTh prepared by the electrochemical method from ACN/TBATFB were also studied. According to Figure 2B, the prepared PDMeBTh shows redox property between 1.17 V vs. SCE (Ea) and 0.63 V vs. SCE (Ec). The oxidation–reduction potential range is basically the same as that of the PDMeBTh prepared from BFEE (Figure 2A). However, the PDMeBTh prepared in BFEE exhibits a wider redox peak, indicating better charge storage capacity and electrochemical activity compared with the PDMeBTh prepared in ACN/TBATFB. In addition, the polymer prepared in ACN/TBATFB was also investigated in concentrated sulfuric acid (Figure 2D). Unfortunately, the peak current density is not proportional to the potential sweep rate, indicating that the polymer has poor redox property in strong acid. At the same time, during CV scanning in concentrated sulfuric acid, the polymer film rapidly degrades and diffuses into the bulk solution. These results may be attributed to the high initial oxidation potential of 3,3′-dimethyl-2,2′-dithiophene in the ACN/TBATFB system, which leads to the degradation of the quality of the prepared PDMeBTh film. Therefore, it can be concluded that the PDMeBTh prepared from BFEE shows better electrochemical properties, which is more suitable for the electrochemical preparation of PDMeBTh compared with the ACN/TBATFB system.

### 2.2. Morphology and Thermal Analysis

**Figure 3:**
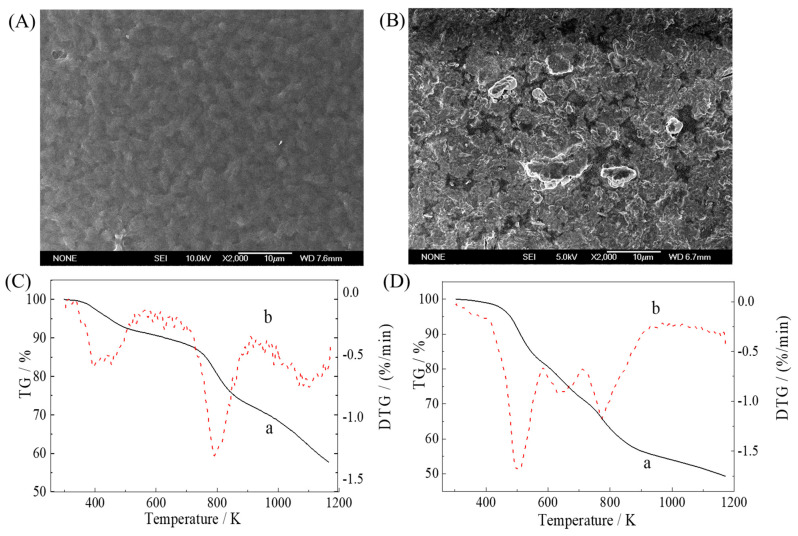
SEM images of the PDMeBTh prepared in (**A**) BFEE and (**B**) ACN/TBATFB; TG (a) and DTG (b) curves of the PDMeBTh prepared in (**C**) BFEE and (**D**) ACN/TBATFB.

The conductivity of PDMeBTh prepared from BFEE and ACN/TBATFB systems are measured to be 5.38 S cm^−1^ and 1.96 S cm^−1^, respectively, which are measured by the four-electrode method. As we all know, the properties of conducting polymers are related to their surface morphologies and structures. The SEM images of PDMeBTh prepared from BFEE and ACN/TBATFB system are illustrated in Figure 3A,B. In comparison, the surface morphology of the PDMeBTh film prepared in BFEE is flatter and denser than that prepared from ACN/TBATFB, which leads to higher conductivity to a certain extent [24,25].

The thermal stability of undoped PDMeBTh prepared from BFEE and ACN/TBATFB system was investigated. Figure 3C shows the thermogravimetric (TG) curve of PDMeBTh prepared from the BFEE system. As shown, the weight loss of PDMeBTh is about 8.6% when the temperature reaches 565 K. This degradation may be attributed to the evaporation of water vapor or the volatilization of other wet substances in the polymer, which is not necessarily related to the change in polymer structure. However, from 722 to 910 K, the PDMeBTh experiences a rapid weight loss of about 18.8%. The corresponding maximum degradation rate is 791 K, which may be caused by the degradation of the polymer skeleton. The results show that PDMeBTh prepared from BFEE has good thermal stability.

In contrast, the degradation of the backbone chain of PDMeBTh prepared from ACN/TBATFB undergoes three rapid and significant weight loss processes: mass loss from 410 to 585 K is 18.3%, loss from 585 to 712 K is 10.4%, and loss from 712 to 938 K is 15.8%. The corresponding maximum degradation rate is at temperatures of 508, 630, and 772 K, respectively (Figure 3D). The multistage degradation in the entire degradation process from 410 to 938 K may be due to the oligomers and polymers with different chain lengths in the PDMeBTh prepared from ACN/TBATFB [26]. In conclusion, the PDMeBTh prepared from the BFEE system has better thermal stability than that prepared from the ACN/TBATFB system.

### 2.3. Electrochromic Properties of PDMeBTh Film

**Figure 4:**
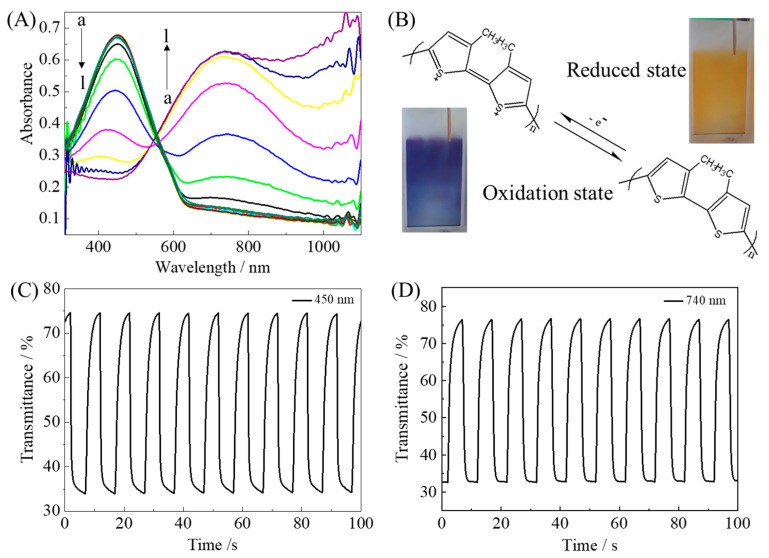
(**A**) Spectroelectrochemistry of the PDMeBTh in ACN/TBATFB under different potentials (V): (a) –1.0, (b) −0.6, (c) −0.4, (d) −0.2, (e) 0.0, (f) 0.2, (g) 0.3, (h) 0.4, (i) 0.5, (j) 0.6, (k) 0.7, and (l) 0.8; (**B**) the molecular structure and photographs of PDMeBTh in oxidation and reduction states; (**C**,**D**) the timed absorption spectra of PDMeBTh at 450 nm and 740 nm.

Spectroelectrochemistry can detect changes in the optical properties of conducting polymers when the voltage changes, which can analyze the data related to the electronic structures of the polymers through the change in energy band during doping. The spectroelectrochemistry of PDMeBTh film was studied in the ACN/TBATFB solution by applying different voltages. The PDMeBTh is in the neutral state at −1.0 V vs. SCE, so only the π-π* transition at 450 nm is shown (Figure 4A). When the voltage gradually reaches 0.8 V vs. SCE, the absorption peak of PDMeBTh at 450 nm gradually disappears, while the absorption peak at 740 nm caused by the polaronic gradually appears and increases to its maximum. As the applied voltage changes, the color of the PDMeBTh film changes from orange-yellow to dark blue, which may be caused by the formation of polaron charges [27]. Figure 4B shows the molecular structure and the colored photographs of PDMeBTh in oxidation and reduction states. The color of PDMeBTh in the reduced state (orange-yellow) is different from the color of poly (3-methylthiophene) (light red) [14]. In the reduced state, the characteristic absorption peak of PDMeBTh is 450 nm, which produces a blue shift of 60 nm compared with the characteristic absorption peak of poly (3-methylthiophene) (510 nm) [14]. This migration of absorption peak may be caused by the introduction of a rotational defect along the polymer chain at a large torsion angle, which interrupts and weakens the π conjugation, thereby affecting the absorption range of light. Hence, the PDMeBTh shows a different color from poly (3-methylthiophene) in the reduced state.

In electrochromic applications, the ability of polymer films to transform quickly and the presence of significant color changes are very important. The electrochromic conversion characteristics of PDMeBTh film were studied by repeated redox processes, and the change in transmittance was detected. In the experiment, the transmittance at the maximum absorption peak of the polymer was measured by an ultraviolet–visible spectrophotometer. The duration of each voltage for the transition between oxidation and reduction states was 5 s. Figure 4C,D show the timed absorption spectra of the PDMeBTh at 450 nm (orange-yellow) and 740 nm (dark blue), respectively. As shown, the optical contrast (ΔT%) between oxidized and reduced states of the polymer is 40.7% at 450 nm and 43.8% at 740 nm. The PDMeBTh at 450 nm has a response time of 1.1 s from the oxidation state to the reduced state and 2.8 s from the reduced state to the oxidation state. The response time from the oxidation state to the reduced state at 740 nm is 3.1 s and from the reduced state to the oxidation state is 1.2 s. The response time is measured based on the time it takes for the optical transmittance to convert to 95%, which are shown in Figure S2A,B. For an electrochromic material, coloring efficiency (CE) is an important parameter to evaluate the electrochromic properties. The maximum CE of PDMeBTh film at 450 nm is 183 cm^2^ C^−1^ and 188 cm^2^ C^−1^ at 740 nm, which can be calculated by combining the data obtained by electrochemical and UV–visible scanning. The CE value is larger than poly(3-methylthiophene) (135 cm^2^ C^−1^) [14], poly(BisTh-o-2F) (70 cm^2^ C^−1^) [28], P(BT4E-DOT) (29 cm^2^ C^−1^) [29], PTh (168 cm^2^ C^−1^) [30], and P6ICA (142 cm^2^ C^−1^) [31]. The prepared PDMeBTh may have potential applications in electrochromic devices due to its good electrochromic performance.

### 2.4. Electrochromic Properties of ECD Constructed with PDMeBTh

**Figure 5:**
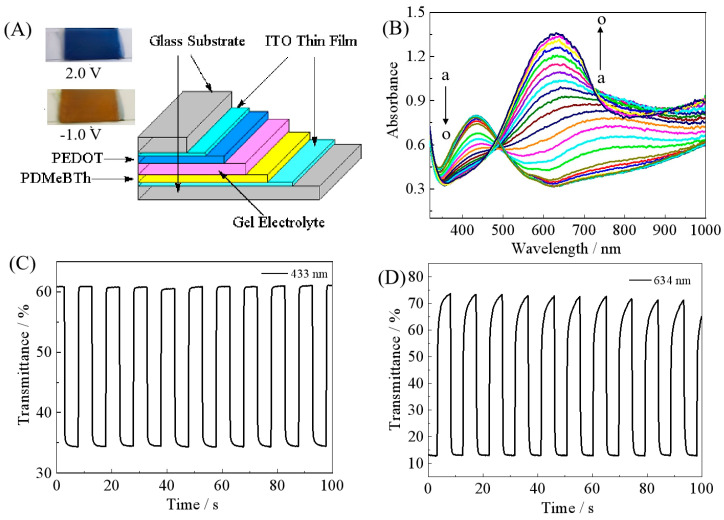
(**A**) The structure of the constructed ECD and its color at different voltages; (**B**) the spectroelectrochemistry of PDMeBTh/PEDOT device under different voltages (V): (a) –1.0, (b) −0.8, (c) −0.6, (d) −0.4, (e) −0.2, (f) 0.2, (g) 0.4, (h) 0.6 (i) 0.8, (j) 1.0, (k) 1.2, (l) 1.4, (m) 1.6, (n) 1.8, and (o) 2.0; (**C**,**D**) the timed absorption spectra of the ECD at 433 nm and 634 nm.

An electrochromic device was constructed with the PDMeBTh and PEDOT due to their good electrochromic properties. The structure of the constructed ECD is shown in Figure 5A. The spectroelectrochemical curve of the PDMeBTh/PEDOT device in the voltage range from −1.0 V to 2.0 V is shown in Figure 5B. When a voltage of −1.0 V is applied, the PDMeBTh is in the neutral state with a π-π* transition of around 433 nm. Meanwhile, PEDOT is in the oxidation state, and its color is transparent light blue in this state. Hence, the color of the device at −1.0 V is orange-yellow dominated by the neutral state of PDMeBTh. The colored photograph of the device in this state is shown in Figure 5A. With the increase in voltage, the absorption peak of PDMeBTh at 433 nm gradually disappears. At the same time, an absorption peak at 634 nm appears, which is caused by the π-π* transition of PEDOT in its neutral state. In this process, the PDMeBTh is gradually oxidized, and PEDOT is gradually reduced. The color of the device also changed from orange-yellow to dark blue due to the superposition of the oxidized state of PDMeBTh (dark blue) and the neutral state of PEDOT (dark blue). The colored photograph of the device in this state is shown in Figure 5A. These results show that the constructed device has good electrochromic performance and an obvious color change.

Figure 5C,D show the timed absorption spectra of PDMeBTh/PEDOT device at 433 nm (A) and 634 nm (B), respectively. The ΔT% of the device is 27% at 433 nm and 61% at 634 nm. At 433 nm, the response time is 0.21 s and 0.29 s, respectively, when the contrast conversion is 95% between the oxidation state and the reduced state. At 634 nm, the response time is 2.17 s and 0.41 s (Figure S2C,D). Combined with the spectroelectrochemical data, the maximum CE of PDMeBTh/PEDOT device can be calculated as 403 cm^2^ C^−1^ at 433 nm and 577 cm^2^ C^−1^ at 634 nm. This CE value is larger than the ECD constructed by poly(3-methylthiophenes) and PEDOT (336 cm^2^ C^−1^) [14] and poly(3,4-dibromothiophene) and PEDOT (406 cm^2^ C^−1^) [32]. Such high CE values of the constructed ECD may be attributed to the good electrochromic properties and coloring ability of PDMeBTh.

### 2.5. Color Memory and Stability of the Device

**Figure 6:**
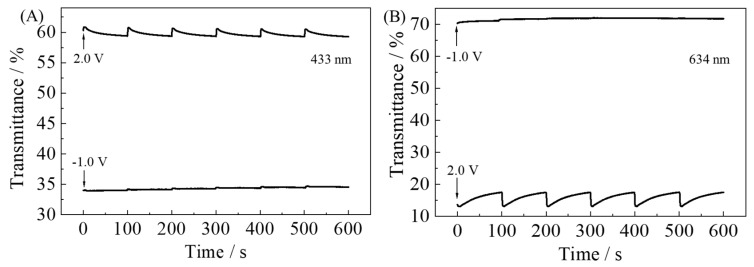
Open circuit memory of ECD at (**A**) 433 nm and (**B**) 634 nm, with applied potentials of −1.0 V and +2.0 V.

In the use of ECDs, the ability to retain color when no additional energy is being supplied is a very important characteristic [33,34]. The spectrum change in the device was measured at 433 nm and 634 nm by applying alternating voltages of −1.0 V and 2.0 V for one second, with a 100 s time interval in between. As can be seen from Figure 6A, both the oxidation state and reduced state of the PDMeBTh/PEDOT device at 433 nm show good color memory ability. The device also has a good color memory at 634 nm, with only a small change in transmittance when no voltage is applied (Figure 6B). This result means that the device does not need to re-apply current to maintain its color in oxidation and neutral states, showing good color memory performance.

**Figure 7:**
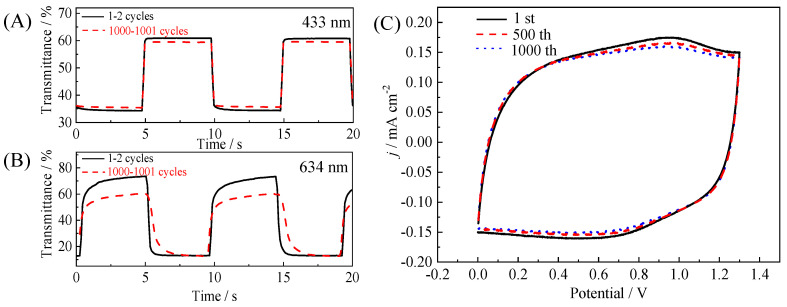
(**A**,**B**) The timed absorption spectra of the ECD for 1–2 cycles and 1000–1001 cycles at 433 nm and 634 nm; (**C**) the redox stability of the ECD.

Long-term transmittance changes are studied to verify the electrochromic stability of the device. Figure 7A,B show the timed absorption spectra of the ECD for 1–2 cycles and 1000–1001 cycles at 433 nm and 634 nm. The transmittance of the device at 433 nm barely changes after 1000 electrochromic cycles. At 643 nm, the transmittance of the device only reduces by approximately 13%. These results imply that the PDMeBTh/PEDOT device has good electrochromic stability. The electrochemical cyclic stability of PDMeBTh/PEDOT devices is also measured by cyclic voltammetry. The device applies voltages between 0.0 V vs. SCE and 1.3 V with a scan rate of 250 mV s^−1^. As shown in Figure 5C, the device can repeat 1000 cycles, while the electrochemical activity only exhibits a small reduction. This indicates that the constructed PDMeBTh/PEDOT device has good electrochemical stability, showcasing its promising practical applications.

## 3. Materials and Methods

The chemical reagents and apparatus used in this study, the details of electrosynthesis of PDMeBTh films, the construction of PDMeBTh/PEDOT electrochromic device, and parameter calculation are all illustrated in the Supporting Information.

## 4. Conclusions

High-performance PDMeBTh with good electrochemical properties and thermal stability has been successfully prepared in BFEE. Due to the effect of large plane distortion angle of the monomer DMeBTh, the prepared PDMeBTh exhibits different color changes compared to the poly (3-methylthiophene), which can be reversibly transformed between orange-yellow and dark blue. The PDMeBTh also shows fast electrochromic response and high coloring efficiency. The color of the constructed PDMeBTh/PEDOT device also can be reversibly changed between orange-yellow and dark blue, which enriches the color of the currently constructed ECD. The fast color response, high optical contrast, high coloring efficiency, good open circuit memory, and cycle stability of the constructed ECD give it good application potential. This research provides a new approach and strategy for regulating the color change in electrochromic materials by adjusting the spatial structure of conducting polymers.

## Data Availability

Data are contained within the article and Supplementary Materials.

## Supplementary Materials

The following supporting information can be downloaded at: https://www.mdpi.com/article/10.3390/molecules29225477/s1, Figure S1: The photographs of the PDMeBTh film polymerized for 10 s at 1.5 V in (A) ACN/TBATFB and 1.0 V in (B) BFEE. Figure S2: The response time for the PDMeBTh film at (A) 450 nm and (B) 740 nm and for the PDMeBTh/PEDOT device at (C) 433 nm and (D) 634 nm.
