# Supplementary material for: Facile Preparation of High-Performance Polythiophene Derivative and Effect of Torsion Angle Between Thiophene Rings on Electrochromic Color Change
Source: Molecules. 2024 Nov 20;29(22):5477. doi: 10.3390/molecules29225477 (PMC11597324; doi:10.3390/molecules29225477)
Supplement: Supplementary file 1 [file molecules-29-05477-s001.zip › molecules-3320949-supplementary.pdf]

Supporting Information

Facile Preparation of High-Performance Polythiophene Derivative and Effect of Torsion Angle Between Thiophene Rings on Electrochromic Color Change

Qingfu Guo [1,*], Chao Sun [2], Yiran Li [1], Kaoxue Li [1] and Xishi Tai [1,*]

[1] College of Chemistry and Chemical Engineering, Weifang University, Weifang 261061, China
[2] Shandong Peninsula Engineering Research Center of Comprehensive Brine Utilization, Weifang University of Science and Technology, Weifang 262700, China
* Correspondence: qfguo@qust.edu.cn (Q.G.); taixs@wfu.edu.cn (X.T.); Tel.: +86-536-8785580 (Q.G.)

Experimental section

1. Materials

BFEE (Beijing Changyang Chemical Plant) was distilled and stored at −20 °C before use. 3,3'-dimethyl-2,2'-bithiophene (Acros Organics, 99%) was used as-received. Tetrabutylammonium tetrafluoroborate (TBATFB, Acros Organics, 95%) was dried in vacuum at 60°C for 24 h before use. Commercial HPLC-grade acetonitrile (ACN, made by Tianjin Guangfu Fine Chemical Research Institute, China) was used directly without further purification. Sulfuric acid (98%) (Jinan Chemical Reagent Company, China) was used as-received.

2. Apparatus

The spectroelectrochemical properties of the polymer were studied by Model 263 electrochemical workstation (EG&G Princeton Applied Research, USA) and Cary 50 UV-Vis spectrophotometer (Varian, USA) under computer control. The thermogravimetric analysis (TGA) was performed with a thermal analyzer of

NETZSCH TG209. Scanning electron microscopy (SEM) measurements were conducted by using a JEOL JSM-6700F scanning electron microscope. CHI 660E electrochemical workstation (Shanghai Chenhua Ins., China) was used to carry out the cyclic voltammetry (CV) measurement of the material and the constructed electrochromic device.

3. Electrosynthesis of PDMeBTh film

Electrochemical synthesis and examinations were performed in a one-compartment cell with the use of a CHI 660E electrochemical workstation under computer control. The working and counter electrodes for cyclic voltammetry experiments were the ITO electrode and platinum wires with a diameter of 0.5 mm placed 0.5 cm apart. To obtain a sufficient amount of polymer for characterization, stainless steel sheets with a surface area of 10 and 12 cm^2 each were employed as the working and counter electrodes, respectively. Electrodes mentioned above were carefully polished with abrasive paper (1500 mesh) and cleaned with water and acetone successively before each examination.

The typical electrolytic solution was BFEE or ACN/TBATFB containing 0.05 mol L^{-1} 3,3’-dimethyl-2,2’-bithiophene. All solutions were deaerated by a dry argon stream and maintained at a slight argon overpressure during experiments. The amount of polymer deposited on the electrode was controlled by the integrated charge passed through the cell. The PDMeBTh film was electrochemically synthesized in BFEE and ACN/TBATFB at a constant potential of 1.0 V *vs.* SCE and 1.5 V *vs.* SCE, respectively. In order to remove the electrolyte and oligomers/monomers, the electropolymerized

films were rinsed with acetone. For TGA, the polymer was dedoped with 25% ammonia for 3 days and then washed repeatedly with water and acetone. Finally, it was dried under vacuum at 60 °C for 2 days.

4. Preparation of gel electrolyte

The gel electrolyte used for ECD construction was prepared by TBATFB, ACN, poly(methyl methacrylate), and polycarbonate in a ratio of 3:70:7:20. TBATFB was dissolved in ACN, and then, poly(methyl methacrylate) was added in this process, with constant stirring and heating. After the poly(methyl methacrylate) is completely dissolved, polycarbonate is added to the reaction system as a plasticizer. The mixture is continuously stirred at 70 ° C until a highly transparent gel electrolyte is formed.

5. The construction of PDMeBTh/PEDOT electrochromic device

The asymmetric electrochromic device had a sandwich structure. The prepared PDMeBTh and PEDOT were coated separately on ITO electrodes. Firstly, PEDOT was prepared in ACN/TBATFB using the constant potential method, and the polymerization voltage was 1.2 V *vs.* SCE. During the polymerization process of PEDOT, the working and counter electrodes were the ITO electrode and stainless steel sheets, and the saturated calomel electrode was used as a reference electrode. Then, the PDMeBTh was prepared in BFEE using the constant potential method. In order to maintain the injected/ejected charge balance, the two electrodes need the same electric quantity during the redox process. Before the device was constructed, the PDMeBTh electrode was in the neutral state, while the PEDOT electrode was in the oxidation state. Finally, the PDMeBTh and PEDOT electrodes were separated by the prepared gel electrolyte.

After constant-temperature drying in an oven at 65 °C, a solid stat device is constructed.

6. Parameter calculation

The optical density (ΔOD) at a specific wavelength (λ_{max}) was determined by using %T values of the electrochemically oxidized and reduced films, using Equation (S1):

$$\Delta OD = \log(T_{ox}/T_{red}) \quad (S1)$$

The coloration efficiency (CE) is defined as the relation between the injected/ejected charge as a function of the electrode area (Q_d) and the change in optical density, ΔOD, at a specific dominant wavelength (λ_{max}) as illustrated by the following Equation (S2):

$$CE = \Delta OD / Q_d \quad (S2)$$

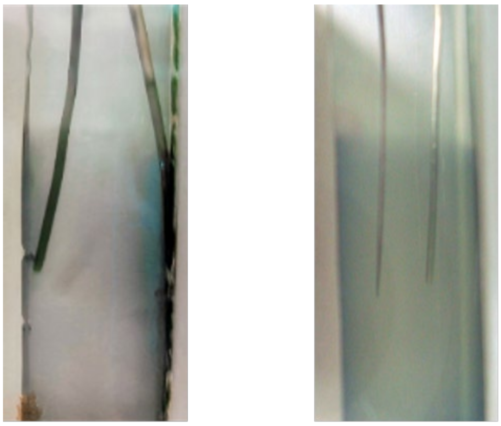

(A) (B)

Figure S1 The photographs of the PDMeBTh film polymerized for 10 s at 1.5 V in (A) ACN/TBATFB and 1.0 V in (B) BFEE.

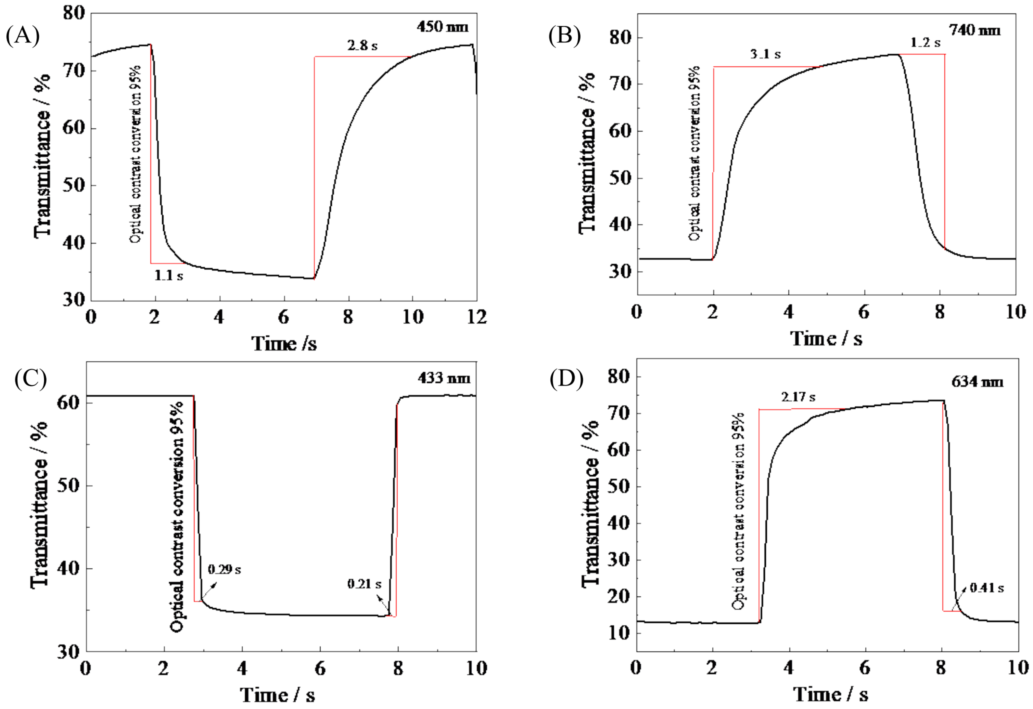

Figure S2 The response time for the PDMeBTh film at (A) 450 nm and (B) 740 nm and for the PDMeBTh/PEDOT device at (C) 433 nm and (D) 634 nm.
